# Methodological Challenges of Emulating a Target Trial to Assess Effectiveness of Timing of PCSK9 Inhibitor Treatment Initiation Post Myocardial Infarction

**DOI:** 10.1002/pds.70354

**Published:** 2026-03-27

**Authors:** Thomas Cars, Stefan Gustafsson, Queenie Chan, Nafeesa Dhalwani, Shia T. Kent, Andrew Briggs, Chris P. Gale, Anselm Gitt, J. Wouter Jukema, Philippe Gabriel Steg, Johan Sundström, Stefan James, Emil Hagström, M. Alan Brookhart

**Affiliations:** ^1^ Sence Research AB Uppsala Sweden; ^2^ Amgen Ltd, Center for Observational Research Cambridge UK; ^3^ Amgen Inc, Center for Observational Research Thousand Oaks California USA; ^4^ London School of Hygiene & Tropical Medicine London UK; ^5^ School of Medicine University of Leeds Leeds UK; ^6^ Department of Cardiology Herzzentrum Ludwigshafen Ludwigshafen am Rhein Germany; ^7^ Department of Cardiology Leiden University Medical Center the Netherlands; ^8^ Université Paris‐Cité, INSERM_1148 Paris France; ^9^ AP‐HP, Hôpital Bichat Paris France; ^10^ Department of Medical Sciences Uppsala University Uppsala Sweden; ^11^ Uppsala Clinical Research Center Uppsala University Uppsala Sweden; ^12^ Department of Population Health Science Duke University Durham North Carolina USA

**Keywords:** clone‐censor‐weight method, immortal time bias, negative control outcomes, trial emulation

## Abstract

**Background:**

Studies comparing treatment strategies based on initiation timing—such as starting PCSK9 inhibitor (PCSK9i) therapy sooner versus later after a myocardial infarction (MI)—are prone to immortal time bias. Clone‐censor‐weight methods can address these issues and allow the researcher to emulate a trial in which patients are assigned to protocols dictating when PCSK9i is initiated. This study aimed to evaluate the comparability of patients in a clone‐censor‐weight setup who initiated a PCSK9i within 12 months post‐MI versus non‐initiators.

**Methods:**

We included adult patients hospitalized for MI in Sweden (2015–2021) and followed them for 3 years. We considered two treatment strategies: initiating PCSK9i within 12 months versus not initiating PCSK9i during the same period. We applied the clone‐censor‐weight method to address immortal time bias and assessed remaining bias using covariate balance metrics and negative control outcomes.

**Results:**

The primary study sample included 38  627 episodes of MI, with 561 (1.5%) initiating PCSK9i treatment within 12 months. These patients were younger, had higher baseline LDL‐C levels, and were more frequently treated with ezetimibe during their post‐MI follow‐up compared to non‐initiators. Although clone‐censor‐weight estimation was free of immortal time bias, it faced challenges in achieving adequate balance of covariates due to the high rates of censoring (relatively small number of people initiating a PCSK9i in the first year) and strong association between covariates and censoring. Truncation of weights provided more stable estimates but at the expense of some covariate imbalances.

**Conclusions:**

The clone‐censor‐weight method is a promising approach that allows researchers to answer questions about the effect of treatment policies. But practical guidance is needed to address problems that arise from small, highly imbalanced groups, which is common with most newly introduced treatments.

## Plain Language Summary

Studies comparing patients who start a medicine at different times after a clinical event can be complicated. Timing often reflects disease duration or severity. For example, after a heart attack, some patients may start cholesterol‐lowering PCSK9 inhibitor treatment early, while others delay or never start. Comparing these groups is difficult because patients must survive long enough to receive the treatment, introducing bias. Early treatment may also only be offered to select patients. Using Swedish healthcare data covering over 38 000 cases of heart attacks between 2015 and 2021, we compared patients who started PCSK9 inhibitors within 12 months to those who did not. We found that very few received early treatment, and they differed in terms of age, low‐density lipoprotein cholesterol levels, and use of another cholesterol‐lowering drug, ezetimibe. We then used a method called clone‐censor‐weighting to help reduce the impact of these differences on health outcomes between treatment groups. We used several methods to check for remaining bias, including looking at unrelated health outcomes that shouldn't be affected by the treatment. Our analysis showed that while the clone‐censor‐weighting method helped reduce bias, it still had challenges when the groups being compared were very different in terms of number of cases and background of patients.

## Key Points

- Clone‐censor‐weight estimation was investigated as a potential strategy to compare two hypothetical treatment strategies: those initiating PCSK9 inhibitors within 12 months post‐myocardial infarction versus those not initiating within the same period.
- The method was unable to achieve adequate balance of covariates in these groups that were highly imbalanced in terms of size and baseline covariates.

## 1 Introduction

Investigations of causal effects of treatment strategies increasingly utilize observational study designs that explicitly emulate randomized clinical trials [1, 2]. A significant challenge in trial emulation arises when comparing strategies based on initiation timing, such as “treat sooner” versus “treat later” after a myocardial infarction (MI). When treatment occurs as a result of an event or clinical decision rather than randomization, it can introduce immortal time bias, as patients must survive long enough to initiate treatment.

Common strategies to mitigate immortal time bias include time‐varying exposure models and landmark analyses [3]. However, both approaches have significant limitations: time‐varying models may inadequately capture dynamic treatment changes, and landmark analyses are prone to selection bias and reduced generalizability due to the arbitrary selection of time points. These limitations are particularly problematic when studying treatments with highly variable and imbalanced initiation patterns, such as proprotein convertase subtilisin/kexin type 9 inhibitors (PCSK9i), where early initiators are often a highly selected subgroup. In Sweden, PCSK9i initiation is further influenced by strict reimbursement criteria, contributing to additional selection bias.

To overcome these challenges, the clone‐censor‐weight (CCW) method presents a promising alternative [4, 5, 6, 7]. This method involves creating identical clones of each participant for each treatment strategy, ensuring that treatment groups are exchangeable at baseline. Participants who deviate from their assigned strategy are censored, and inverse‐probability‐of‐censoring weights are applied to re‐weight the remaining participants. This process maintains conditional exchangeability, effectively reducing immortal time bias in studies where follow‐up does not coincide with treatment initiation.

Despite its potential, the CCW method has seen limited application, and its strengths and limitations need to be further explored. Comparative effectiveness studies of newly introduced treatments typically feature imbalanced groups with few initiators initially, raising questions about the utility of clone‐censor‐weighting in such settings. Additionally, evaluating residual bias through negative control outcomes (NCOs) is essential to assess the effectiveness of the applied methodological strategies before conducting the primary cardiovascular outcomes analysis.

In this study, we investigated whether the trial emulation framework using CCW could establish an unbiased analytical framework to compare early initiation versus non‐ or late initiation of PCSK9i post‐MI. By employing NCOs, we aimed to evaluate residual bias and assess the comparability of initiators and non‐initiators within this methodological context.

## 2 Methods

### 2.1 Data Sources

This retrospective cohort study utilized data collected in Sweden from both regional [8] and national healthcare registries [9] linked with electronic medical records (EMR) [10, 11]. The national data sources provided nearly complete patient coverage as Swedish residents have universal access to health care with a negligible copayment for healthcare visits, hospitalizations, and medications [12]. EMR data were gathered from five regions, representing over half of the Swedish population. The linked data included comprehensive information on inpatient and outpatient diagnoses, clinical procedure codes, drug utilization, causes of death, clinical measurements, and laboratory test results. This linkage was made possible using the personal identity number (PIN) given to each Swedish resident [13]. Detailed descriptions of the data sources are provided in Supporting Information.

### 2.2 Study Sample and Eligibility Criteria

We initially identified all MI diagnoses (ICD‐10 codes I21 and I22) recorded between January 1, 2015, and December 31, 2021. To be included, the MI diagnosis had to be listed as the primary diagnosis in inpatient care, and patients needed to be older than 18 years at the time of the MI. The index date was defined as the discharge date from the MI hospitalization. Only MI cases meeting the following inclusion criteria were retained: a minimum of 12 months of data prior to the MI, at least 1 day of follow‐up after discharge, and no prior treatment with a PCSK9i (ATC codes: C10AX13 and C10AX14). For patients with multiple MI records, we considered diagnoses recorded within 30 days of a previous diagnosis as part of the same MI episode.

To fulfil the positivity assumption (the conditional probability of receiving a treatment cannot be 0 or 1 in any patient subgroup defined by combinations of covariate values), we a priori restricted the study population to patients who were candidates for a PCSK9i by meeting the following criteria: (a) treatment with statins up until MI discharge, (b) at least one low‐density lipoprotein cholesterol (LDL‐C) measurement within 2 years prior to and up until MI discharge, (c) age ≤ 85 years, (d) no history of LDL‐C apheresis, and (e) no recordings of cognitive disorders.

Since LDL‐C measurement data were extracted from five specific Swedish regions, the primary study sample focused on patients from these regions. We also devised a secondary study sample, including patients from all regions of Sweden; we performed sensitivity analyses using this sample employing a modified model to account for the absence of LDL‐C data in regions outside the five specified regions.

### 2.3 Comparison of Treatment Strategies

We compared two treatment strategies: initiating treatment with PCSK9i within 12 months following a MI (early PCSK9i) versus not initiating PCSK9i treatment within the same period (no or late PCSK9i). We used two methods for assessing residual bias in the analytical framework: investigating rates of NCOs and investigating balance in baseline covariates.

### 2.4 Negative Control Outcomes

An NCO is an outcome variable expected to be causally unrelated to the exposure, making it a useful tool for assessing potential bias in the analytical framework. Using subject matter knowledge, the NCOs that we selected for this study included bone fractures, urethritis, hip and/or knee arthroplasty, kidney stones, glaucoma, non‐melanoma skin cancer, and other cancers. To avoid prevalent cases, the assessment of cancer‐related NCOs, kidney stones, and glaucoma was restricted to individuals with no prior history of these conditions, based on all available data. For all other NCOs, the sample was restricted to individuals without a recorded diagnosis of the condition within 90 days before the index date. Details on the NCO definitions, including the specific codes used, are provided in the Supporting Information.

### 2.5 Follow‐Up

Each MI episode was followed for 3 years and censored at time to first negative control outcomes, death, loss to follow‐up, or whichever came first. Detailed definitions of outcomes are provided in Supporting Information.

### 2.6 Covariates

Covariates included age, sex, chronic kidney disease (CKD), heart failure (HF), atherosclerotic cardiovascular disease (ASCVD), dyslipidemia, hypertension, diabetes mellitus, obesity, muscle‐related disorders, pancreatitis, hepatic disease, chronic obstructive pulmonary disease (COPD), treatment with statins and their intensity, treatment with ezetimibe, level of low‐density lipoprotein cholesterol (LDL‐C), residency, and calendar period. Detailed definitions of these covariates are provided in Supporting Information.

### 2.7 Statistical Analysis

To emulate a target trial, we applied a framework of cloning, censoring, and weighting in the study [4, 5, 6]. For each MI episode, two clones (copies) were created and assigned to each treatment strategy: early PCSK9i and no (or late) PCSK9i. Each clone was assumed to comply with its respective treatment strategy at the index date and to have some probability of receiving PCSK9i during the first year of follow‐up. At weekly intervals, we assessed whether the clones adhered to their assigned treatment strategy. If a clone deviated from the assigned treatment strategy, it was censored. For example, if a clone assigned to not initiate PCSK9i within 12 months started treatment at 6 months, it would be censored at that time point (informative censoring). To adjust for potential selection bias induced by this informative censoring, two weight models for the probability of informative censoring were fitted, reflecting the asymmetric censoring mechanisms in the two strategies [6]. In the early PCSK9i arm, no informative censoring occurs during the 12‐month grace period, as clones do not deviate from this treatment strategy as long as they have the opportunity to initiate treatment. Censoring occurs only at month 12 for those who did not initiate, and a logistic regression model was fitted at this time point. In the no (or late) PCSK9i arm, clones are censored upon treatment initiation, which can occur at any weekly interval. A pooled logistic regression model was therefore fitted for the censoring at each interval, including time‐updated covariates at the start of each interval.

The model using the primary study sample included the following baseline covariates: age (modeled as a restricted cubic spline with 3 knots), sex, CKD, HF, hypertension, muscle‐related disorders, pancreatitis, hepatic disease, diabetes mellitus, obesity, ASCVD, dyslipidemia, statin intensity, treatment with ezetimibe, LDL‐C (modeled as a restricted cubic spline with 3 knots), and calendar period. Additionally, the model included the following time‐varying covariates: statin intensity, treatment with ezetimibe, LDL‐C (modeled as a restricted cubic spline with 3 knots), stroke, and subsequent MI. Definitions of covariates and formula for the statistical models are presented in Supporting Information.

The cumulative probability of a clone remaining uncensored up until the start of a given time segment was extracted from the fitted models for each treatment strategy, and inverse probability of censoring weights (IPCW) of remaining uncensored were calculated. Because no informative censoring occurs in the early PCSK9i arm during the 12‐month period, weights were by definition equal to 1 up until the end of the first year.

Weights calculated at the end of the informative censoring period (i.e., at 12 months) were retained for the remainder of the follow‐up. Extreme weights greater than the 99.9th percentile of all weights per strategy were capped at the 99.9th percentile value [14, 15]. The distribution of weights and effective sample sizes (ESS), calculated as ESS = (Σ*wᵢ*)^2^/Σ*wᵢ* ^2^, are reported in Table S1. In a sensitivity analysis, the model was also evaluated for the NCO fracture using the original, non‐truncated weights. Confidence intervals (95%) were estimated using standard errors from a nonparametric bootstrap procedure with 500 replications [16].

Covariate balance between the two treatment strategies was assessed as standardized mean differences at a single time point at the first week after the first year of follow‐up, that is when all informative censoring events had occurred.

Analyses were performed according to the intention‐to‐treat (ITT) strategy, with follow‐up truncated at 3 years. The ITT framework was applied because the research question focuses on comparing the strategies “treat early” versus “treat later,” where patients are assigned to treatment groups based solely on the timing of their initiation. This approach does not account for subsequent treatment discontinuation, as doing so would shift the focus to also include treatment persistence, making the analysis less clinically intuitive.

We conducted two sensitivity analyses. The first was a sensitivity analysis without LDL‐C, as LDL‐C may be viewed as a mediator. This analysis used the secondary sample including patients from all regions of Sweden, of which most lacked LDL‐C data. The model used for this analysis was similar to the main model, excluding all LDL‐C variables. The second sensitivity analysis was designed to determine whether similar covariate balance between the early PCSK9i and no (or late) PCSK9i groups could be achieved using a less complex statistical framework, compared to the CCW approach. For this analysis, we developed a more traditional propensity score‐weighted model. Here, we restricted the primary study sample to patients with at least 12 months of follow‐up after MI discharge (i.e., all patients survived for at least 12 months). Patients were categorized into the early PCSK9i group if they initiated PCSK9i treatment within 12 months post‐index. All analyses were performed using R statistical software (version 3.6.0) [17].

## 3 Results

**FIGURE 1:**
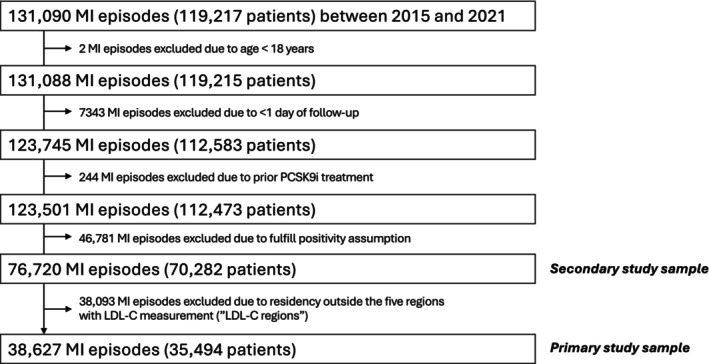
Flow‐chart of the derivation of the primary and secondary study samples. LDL‐C, low‐density lipoprotein cholesterol; MI, myocardial infarction; PCSK9i, proprotein convertase subtilisin/kexin type 9 inhibitor.

The derivation of the study sample is outlined in Figure 1, and baseline characteristics are presented in Table 1. In the primary study sample, 38 627 MI episodes from 35 494 patients were cloned into both treatment strategies. Among these, 561 patients (1.5%) initiated PCSK9i treatment within 12 months post‐MI. Compared with no (or late) PCSK9i initiators, these early initiators were significantly younger (median [SD] age 65.1 [10.3] vs. 68.8 [10.3] years), had higher baseline LDL‐C levels (median LDL‐C 3.7 [1.6] vs. 2.7 [1.2] mmol/L), and were more frequently treated with ezetimibe at discharge (6.1% vs. 0.9%) (Table 1).

**TABLE 1:** Baseline characteristics for the primary and secondary study samples.

	Primary study sample			Secondary study sample
	Total	Early PCSK9i	No (or late) PCSK9i	Total
*N*	38 627	561	38 066	76 720
Age at index (mean, SD)	68.8 (10.3)	65.1 (10.3)	68.8 (10.3)	69.5 (10.2)
Female (*N*, %)	11 190 (29.0)	223 (39.8)	10 967 (28.8)	23 097 (30.1)
Baseline LDL‐C, mmol/L (mean, SD)	2.7 (1.2)	3.7 (1.6)	2.7 (1.2)	2.8 (1.2)
Comorbid conditions (*N*, %)				
ASCVD	19 553 (50.6)	286 (51.0)	19 267 (50.6)	39 446 (51.4)
Dyslipidemia	19 195 (49.7)	386 (68.8)	18 809 (49.4)	34 788 (45.3)
Heart failure	8614 (22.3)	80 (14.3)	8534 (22.4)	17 445 (22.7)
Chronic kidney disease	4949 (12.8)	56 (10.0)	4893 (12.9)	9136 (11.9)
Diabetes mellitus	12 503 (32.4)	145 (25.8)	12 358 (32.5)	24 614 (32.1)
Hypertension	27 246 (70.5)	386 (68.8)	26 860 (70.6)	54 168 (70.6)
Obesity	5135 (13.3)	97 (17.3)	5038 (13.2)	8239 (10.7)
Muscle related disorders	927 (2.4)	25 (4.5)	902 (2.4)	1607 (2.1)
Pancreatitis	666 (1.7)	10 (1.8)	656 (1.7)	1349 (1.8)
Hepatic disease	1116 (2.9)	28 (5.0)	1088 (2.9)	1983 (2.6)
COPD	3889 (10.1)	44 (7.8)	3845 (10.1)	7385 (9.6)
Lipid lowering treatments (at baseline) (*N*, %)				
Statin and ezetimibe	2455 (6.4)	110 (19.6)	2345 (6.2)	4427 (5.8)
Statin only	31 019 (80.3)	288 (51.3)	30 731 (80.7)	61 165 (79.7)
Ezetimibe only	391 (1.0)	34 (6.1)	357 (0.9)	811 (1.1)

Abbreviations: ASCVD, atherosclerotic cardiovascular disease; COPD, chronic obstructive pulmonary disease; LDL‐C, low‐density lipoprotein cholesterol; PCSK9i, proprotein convertase subtilisin/kexin type 9 inhibitors.

**FIGURE 2:**
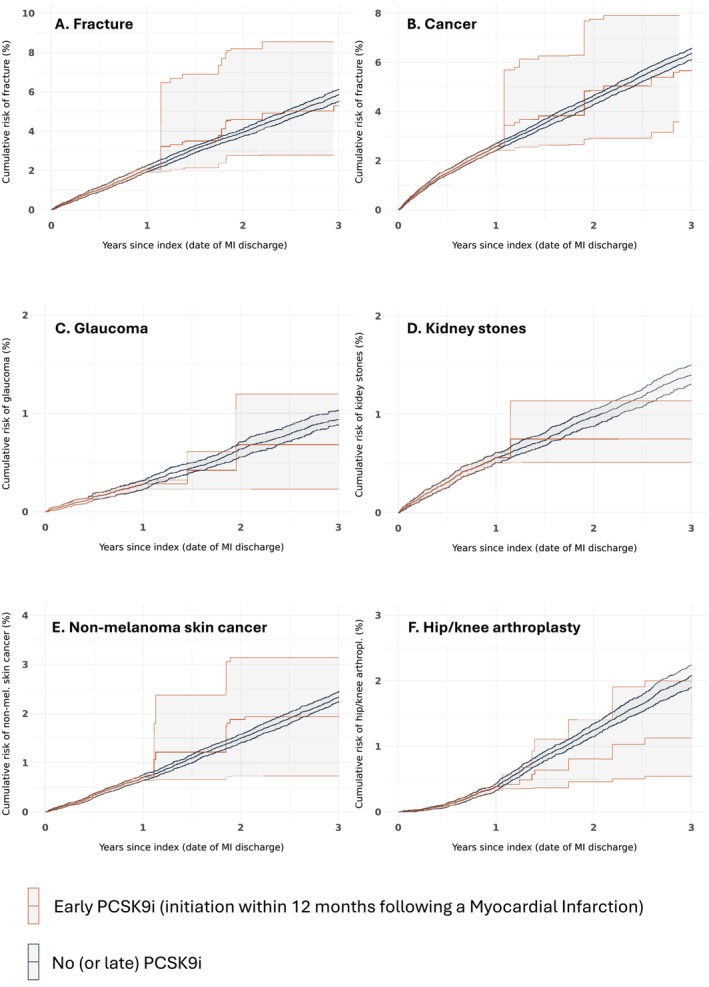
Weighted Kaplan–Meier estimates for the negative control outcomes in the early versus no (or late) PCSK9i treatment strategies. PCSK9i, proprotein convertase subtilisin/kexin type 9 inhibitor.

**FIGURE 3:**
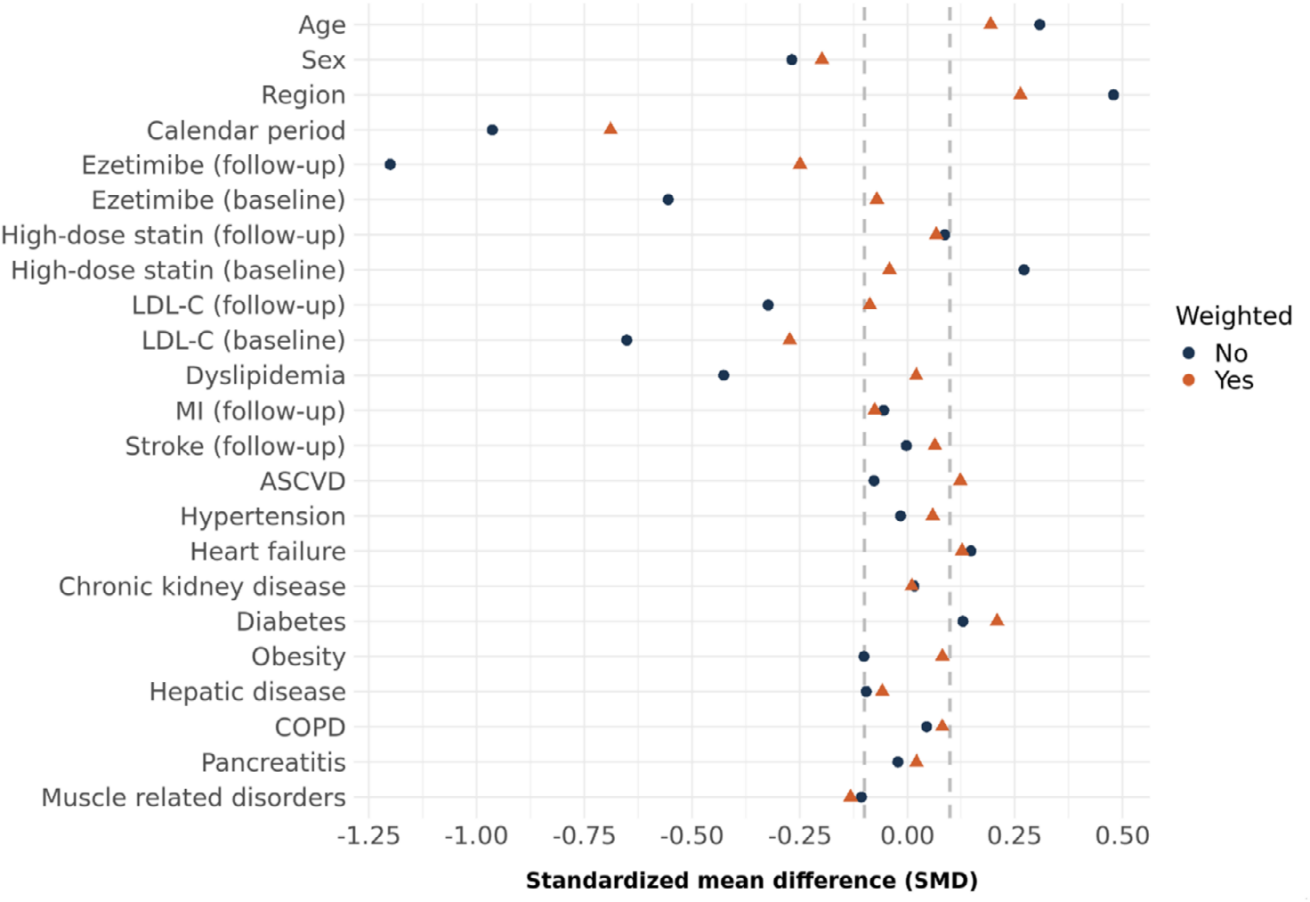
Assessment of covariate balance for the fracture outcome in the primary study sample. ASCVD, atherosclerotic cardiovascular disease; COPD, chronic obstructive pulmonary disease; LDL‐C, low‐density lipoprotein cholesterol; MI, myocardial infarction.

Over the three‐year study period, bone fractures and cancer exhibited the highest incidence rates among all NCOs (Figure S1). Effect estimates comparing early versus no (or late) PCSK9i treatment strategies in the primary study population are detailed in Table 2 and Figure 2 (Panels A–F). For several NCOs, particularly those with higher incidences such as bone fractures and cancer, the treatment effects were neutral, with hazard ratios (HR) close to 1 (HR for fractures = 0.96, 95% CI: 0.62–1.65; HR for cancer = 0.94, 95% CI: 0.66–1.35) (Table 2). However, non‐neutral effects were observed for arthroplasty (HR = 0.62, 95% CI: 0.38–1.01) and kidney stones (HR = 0.84, 95% CI: 0.75–0.98). As there is no plausible biological mechanism linking PCSK9i treatment to these outcomes, these findings are likely a result of residual confounding, consistent with the covariate imbalances observed for LDL‐C values and ezetimibe use (Figure 3).

**TABLE 2:** Effects of the early versus no (or late) PCSK9i treatment strategies on the negative control outcomes.

	Hazard ratio	Risk difference (%) (at 3 years)
Bone fracture	0.96 (95% CI: 0.62–1.65)	−0.57 (95% CI: −2.75; 4.03)
Cancer	0.94 (95% CI: 0.66–1.35)	−0.70 (95% CI: −2.73; 1.65)
Arthroplasty	0.62 (95% CI: 0.38–1.01)	−0.95 (95% CI: −1.59; −0.12)
Kidney stone	0.84 (95% CI: 0.75–0.98)	−0.65 (95% CI: −0.91; −0.25)
Glaucoma	0.79 (95% CI: 0.40–1.23)	−0.26 (95% CI: −0.69; 0.23)
Non melanoma skin cancer	0.90 (95% CI: 0.43–1.41)	−0.41 (95% CI: −1.66; 0.79)

Although untruncated weights slightly improved covariate balance, this was accompanied by a reduction in effective sample size, particularly in the early PCSK9i arm. Covariate balance for the primary truncation level (99.9th percentile) is shown in Figure S2, and the progression of covariate balance over the censoring period is shown in Figure S5.

Sensitivity analyses produced similar results to the main analyses. Results in the primary study samples were consistent with those in the broader secondary study sample (Figure 1 and Figure S3). When restricting the sample to patients with at least 12 months of follow‐up after MI discharge, the sensitivity analysis of this less complex model still exhibited covariate imbalance issues similar to the primary models (Figure S4).

## 4 Discussion

The CCW method [5] is a promising approach for estimating the causal effects of treatment protocols. It mitigates immortal time bias and addresses other sources of bias, including baseline confounding and informative censoring due to time‐varying factors. We assessed the performance of a CCW estimator in a study comparing a rarely and selectively followed protocol—early initiation of a PCSK9i—against non‐ or late initiators following MI, using several NCOs. Our findings suggest that, in this specific context, the method struggled to achieve adequate covariate balance and produced variable risk estimates.

By using covariate balance assessments and NCOs to evaluate residual bias, we observed that the CCW method struggled to balance groups that were highly imbalanced in terms of size and baseline covariates. This challenge is particularly common in studies of newly introduced treatments, where early adopters and late or non‐initiators may differ substantially. The challenges were particularly pronounced for rare outcomes, where censoring weights can become extreme, leading to unreliable results. A common approach to mitigate extreme weights is truncation; however, this can introduce additional residual bias [18]. We explored multiple weight truncation strategies and found that the choice of truncation level involved a trade‐off between covariate balance and effective sample size, consistent with the known bias‐variance trade‐off inherent to inverse probability weighting in settings with small and highly selected treatment groups [14, 15, 18]. The truncation sensitivity analysis (Table S2) was conducted using a single representative NCO, as the purpose of this analysis was to identify the optimal truncation level based on the trade‐off between covariate balance and effective sample size, rather than to evaluate residual bias per se. Based on these observations, we concluded that in our data, truncation at the 99.9th percentile provided the best trade‐off between covariate balance and preserving sufficient effective sample size for meaningful inference (Table S2).

One unanswered question about the CCW method is when to evaluate balance. As its main forte is in “treat sooner” versus “treat later” type research questions, as it has the potential to reduce immortal time bias problems, it would seem natural to check for balance after the “treat sooner” time period has ended. We chose that strategy here. The distributions of person‐time until censoring look very different in the two groups, and choosing optimal models to calculate weights is not straightforward. Different model types in the two groups are possible, and with very nearly all in the “treat sooner” group being censored on Day 365 (the new treatment was unusual, leading to highly imbalanced groups), a logistic model could be used while a Cox model or pooled logistic regression could be more natural in the “treat later” group. Further, the choice of defining the groups based on treatment before the specific Day 365 may be discussed; such strategies will have to be formulated with knowledge of the biological systems under study. And as with all comparative effectiveness methods, it is likely that these groups differ in ways that are unmeasured or unmeasurable.

In addition to investigating multiple strategies for modelling and truncating weights, and using multiple qualitatively different NCOs to study balance, we performed multiple sensitivity analyses in different samples, using different model specifications, and using different handling of censoring. These all produced similar results, with similar residual imbalances. Notably, the sensitivity analysis of a simpler model not using the CCW method had similar problems with residual covariate imbalances, indicating that much of the problems observed in the main analysis are due to small sample sizes and large baseline imbalances, rather than the CCW method itself. In the CCW framework, cloning ensures perfect covariate balance at baseline; imbalance emerges progressively as clones are censored for deviating from their assigned strategy, with the majority occurring at the end of the treatment initiation window (Figure S5). We believe that transparent reporting of these dynamics, combined with the use of negative control outcomes and covariate balance diagnostics as demonstrated here, can serve as a practical reference for researchers considering similar analyses of newly introduced treatments.

## 5 Conclusions

In summary, the CCW method is a promising approach for addressing immortal time bias, but careful evaluation of positivity conditions and weight diagnostics is essential, particularly in settings with highly imbalanced groups, which is common with newly introduced treatments.

## Funding

This study was funded by Amgen Inc.

## Ethics Statement

The study was approved by the Swedish Ethical Review Authority (#2021–06817‐02).

## Consent

The authors have nothing to report.

## Conflicts of Interest

T.C. is an employee and shareholder of Sence Research A.B. S.G. is a former employee of Sence Research A.B. Q.C., N.D., and S.T.K. are Amgen employees/shareholders. A.B. serves on scientific advisory committees for AbbVie, Accompany Health, American Academy of Allergy, Asthma & Immunology, Amgen, Axsome Therapeutics, Brigham and Women's Hospital, Gilead/Kite, Merck, National Institute of Diabetes and Digestive and Kidney Diseases, Regeneron, and Target RWE; he owns equity in Accompany Health and VitriVax. C.P.G. has received research grants from Alan Turing Institute, British Heart Foundation, National Institute for Health Research, Horizon 2020, Abbott Diabetes, Bristol Myers Squibb, and European Society of Cardiology; honoraria from, Amgen, AstraZeneca, Bayer, Boehringer‐Ingleheim, Boston Scientific, Bristol Myers Squibb, Chiesi, Daiichi Sankyo, GPRI Research B.V., iRhythm, Menarini, Novartis, Organon, Raisio Group, The Phoenix Group, Wondr Medical, and Zydus; receipt of equipment from Kosmos device. He owns equity in CardioMatics. A.G. has received honoraria from Amgen, Astra Zeneca, Boehringer‐Ingelheim, Daiichi Sankyo, NovoNordisk, and Pfizer; and support for attending meetings and/or travel for NovoNordisk; serves on scientific advisory committees for Amgen. J.W.J. was speaker (with or without lecture fees) at (continuing medical education accredited) meetings sponsored by, and/or his department has received research grants from among others Amgen, Athera, Astra‐Zeneca, Biotronik, Boston Scientific, Dalcor, Daiichi Sankyo, Lilly, Medtronic, Merck‐Schering‐Plough, Pfizer, Roche, Sanofi Aventis, The Medicine Company, the Netherlands Heart Foundation, Cardio Vascular Research the Netherlands (CVON), the Netherlands Heart Institute, and the European Community Framework KP7 Programme. P.G.S. reports Research grants from Amarin, Sanofi, Clinical Trials work for Amarin, Amgen, AstraZeneca, Bayer, Bristol‐Myers Squibb, Idorsia, Janssen, Lilly, Merck, Novartis, Novo‐Nordisk, Pfizer, Sanofi; Consulting or speaking: Amarin, Amgen, BMS, Novo‐Nordisk, Sanofi. He is Chief Medical Officer: Bioquantis and Senior Associate Editor at *Circulation*. J.S. has stock ownership in companies (Anagram kommunikation AB, Sence Research AB, Symptoms Europe AB, MinForskning AB) providing services to companies and authorities in the health sector including Amgen, AstraZeneca, Bayer, Boehringer, Eli Lilly, Gilead, GSK, Göteborg University, Itrim, Ipsen, Janssen, Karolinska Institutet, LIF, Linköping University, Novo Nordisk, Parexel, Pfizer, Region Stockholm, Region Uppsala, Sanofi, STRAMA, Takeda, TLV, Uppsala University, Vifor Pharma, WeMind. S.J. has received institutional grants from AstraZeneca, Novartis, Bayer, Janssen, Amgen, and Chiesi, proctoring fees from Medtronic, and participation on the data safety and monitoring board for studies conducted by New Amsterdam Pharma. E.H. has received institutional research grants from NovoNordisk, Sanofi, Pfizer, and Amgen, and consulting or honoraria from Amgen, Sanofi, Novartis, Pfizer, Amarin, Ultragenyx, and Novo Nordisk. M.A.B. serves on scientific advisory committees for Amgen, Atara Biotherapeutics, National Institute of Diabetes and Digestive and Kidney Diseases, Gilead, Kite, and Target RWE; he owns equity in VitriVax and AccompanyHealth.

## Supporting information


**Data S1:** pds70354‐sup‐0001‐supinfo.docx.
**Figure S1:** Incidence of the six negative control outcomes.
**Figure S2:** Comparison in covariate balance between truncated and non‐truncated weights for the NCO of fractures.
**Figure S3:** Effect estimates and covariate balance for the NCO: fracture in the secondary study sample.
**Figure S4:** Sensitivity analysis using a simpler propensity score‐weighted model.
**Figure S5:** Standardized mean differences between the early PCSK9i and no/late PCSK9i treatment strategies at 0, 3, 6, 9, and 12 months after index, with and without inverse probability of censoring weighting (weights truncated at the 99.9th percentile).
**Table S1:** Distribution of inverse probability of censoring weights and effective sample size by treatment strategy, with and without truncation at the 99.9th percentile.
**Table S2:** Bias‐variance trade‐off across weight truncation levels for the negative control outcome bone fracture.
