# Supplementary material for: Methodological Challenges of Emulating a Target Trial to Assess Effectiveness of Timing of PCSK9 Inhibitor Treatment Initiation Post Myocardial Infarction
Source: Pharmacoepidemiol Drug Saf. 2026 Mar 27;35(4):e70354. doi: 10.1002/pds.70354 (PMC13031884; doi:10.1002/pds.70354)
Supplement: Supplementary file 1 — Data S1: pds70354‐sup‐0001‐supinfo.docx. Figure S1: Incidence of the six negative control outcomes. Figure S2: Comparison in covariate balance between truncated and non‐truncated weights for the NCO of fractures. Figure S3: Effect estimates and covariate balance for the NCO: fracture in the secondary study sample. Figure S4: Sensitivity analysis using a simpler propensity score‐weighted model. Figure S5: Standardized mean differences between the early PCSK9i and no/late PCSK9i treatment strategies at 0, 3, 6, 9, and 12 months after index, with and without inverse probability of censoring weighting (weights truncated at the 99.9th percentile). Table S1: Distribution of inverse probability of censoring weights and effective sample size by treatment strategy, with and without truncation at the 99.9th percentile. Table S2: Bias‐variance trade‐off across weight truncation levels for the negative control outcome bone fracture. [file PDS-35-e70354-s001.docx]

# Supplementary Methods

## Data sources

The following data sources were utilized in this study:

### National Patient Register (NPR)

In 1964, the Swedish National Board of Health and Welfare (NBHW) started to collect data on hospital discharge diagnoses in the National Patient Register (NPR). In 1984, participation became mandatory for all county councils, and in 1987, all inpatient care in Sweden was included. More than 99% of hospital stays are registered in the NPR. Since 2001, NPR has also covered outpatient visits from Swedish hospitals and other specialist clinics in ambulatory care. Information from primary care is not yet included. Data in the NPR include personal identification number (PIN), patient demographics, caregiver information, date of admission/discharge or visit, diagnosis (primary and secondary), and clinical procedures. Diagnoses are coded according to the current version of the International Classification of Disease (ICD). When data are entered into the NPR, checks are performed to secure the quality of the data.

### Prescribed Drug Register (SPDR)

The Swedish prescribed drug register (SPDR) at the NBHW holds information on dispensed drugs on an individual level since July 2005. The SPDR includes information on the dispensed unit including drug identification number, number of dispensed units, costs, patient demographics (including age and sex), date of prescription and dispensation and code for caregiver. Drugs are classified according to the Anatomical Therapeutic Chemical (ATC) classification system. Population coverage in SPDR is estimated to be over 99%. The register does not include data on over-the-counter medications and coverage is not complete for vaccines and drugs used in nursing homes. The SPDR is updated monthly with data from Swedish eHealth Agency.

### Cause of Death Register

The Swedish Cause of Death Register at the NBHW includes information on the date of death and underlying causes for all deaths occurring among Swedish residents recorded since 1961.

### Electronic Medical Records (EMR)

Electronic Medical Record (EMR) information was included in the study from the following five regions in Sweden, covering more than 50% of the Swedish population: Stockholm (24%), Västra Götaland (16.7%), Uppsala (3.5%), Dalarna (2.8%), and Skåne (13.3%). These five regions are referred to as *LDL-regions* in this report (LDL-C measurements have been extracted from these regions).

## Negative Control Outcomes (NCOs)

The following definitions were used to identify NCOs:

### Fractures

| **Code** | **Description** |
| --- | --- |
| M48.5 | Collapsed vertebra, not elsewhere classified |
| M80.0 | Postmenopausal osteoporosis with pathological fracture |
| M80.8 | Other osteoporosis with pathological fracture |
| M84.4 | Pathological fracture, not elsewhere classified |
| M84.5 | Pathological fracture in neoplastic disease |
| M84.6 | Pathological fracture in other disease |
| M84.7 | Nontraumatic fracture, not elsewhere classified |
| S12.0 | Fracture of first cervical vertebra |
| S12.1 | Fracture of second cervical vertebra |
| S12.2 | Fracture of other specified cervical vertebra |
| S12.3 | Fracture of fourth cervical vertebra |
| S12.4 | Fracture of fifth cervical vertebra |
| S12.5 | Fracture of sixth cervical vertebra |
| S12.6 | Fracture of seventh cervical vertebra |
| S12.8 | Fracture of other parts of neck |
| S12.9 | Fracture of neck, part unspecified |
| S22.0 | Fracture of thoracic vertebra |
| S32.0 | Fracture of lumbar vertebra |
| S32.1 | Fracture of sacrum |
| S32.2 | Fracture of coccyx |
| S32.3 | Fracture of ilium |
| S32.4 | Fracture of acetabulum |
| S32.5 | Fracture of pubis |
| S32.6 | Fracture of ischium |
| S32.8 | Fracture of other and unspecified parts of lumbar spine and pelvis |
| S32.9 | Fracture of unspecified parts of lumbosacral spine and pelvis |
| S42.0 | Fracture of clavicle |
| S42.2 | Fracture of upper end of humerus |
| S42.3 | Fracture of shaft of humerus |
| S42.4 | Fracture of lower end of humerus |
| S42.9 | Fracture of shoulder girdle, part unspecified |
| S49.0 | Physeal fracture of upper end of humerus |
| S49.1 | Physeal fracture of lower end of humerus |
| S52.0 | Fracture of upper end of ulna |
| S52.1 | Fracture of upper end of radius |
| S52.2 | Fracture of shaft of ulna |
| S52.3 | Fracture of shaft of radius |
| S52.5 | Fracture of lower end of radius |
| S52.6 | Fracture of lower end of both ulna and radius |
| S52.9 | Fracture of forearm, part unspecified |
| S59.0 | Physeal fracture of lower end of ulna |
| S59.1 | Physeal fracture of upper end of radius |
| S59.2 | Physeal fracture of lower end of radius |
| S72.0 | Fracture of head and neck of femur |
| S72.1 | Pertrochanteric fracture |
| S72.2 | Subtrochanteric fracture |
| S72.3 | Fracture of shaft of femur |
| S72.4 | Fracture of lower end of femur |
| S72.8 | Fractures of other parts of femur |
| S72.9 | Fracture of femur, part unspecified |
| S79.0 | Physeal fracture of upper end of femur |
| S79.1 | Physeal fracture of lower end of femur |
| S82.1 | Fracture of upper end of tibia |
| S82.2 | Fracture of shaft of tibia |
| S82.3 | Fracture of lower end of tibia |
| S82.4 | Fracture of fibula alone |
| S82.5 | Fracture of medial malleolus |
| S82.6 | Fracture of lateral malleolus |
| S82.8 | Fractures of other parts of lower leg |
| S82.9 | Fracture of lower leg, part unspecified |

### Arthroplasty

| **ICD10** | **Description** |
| --- | --- |
| Z966F | Presence of artificial hip joint |
| Z966G | Presence of artificial knee joint |
| NFB (procedure code) | Primary prosthetic replacement of hip joint |
| NGB (procedure code) | Primary prosthetic replacement of knee joint |

### Kidney Stones

| **ICD10** | **Description** |
| --- | --- |
| N20 | Calculus of kidney and ureter |
| N21 | Calculus of lower urinary tract |

### Glaucoma

| **ICD10** | **Description** |
| --- | --- |
| H40.1 | Primary open-angle glaucoma |
| H40.2 | Primary angle-closure glaucoma |
| H40.5 | Glaucoma secondary to other eye disorders |
| H40.9 | Glaucoma, unspecified |

### Non-Melanoma Skin Cancer

| **ICD10** | **Description** |
| --- | --- |
| C44 | Other malignant neoplasms of skin |

### Cancer

| **ICD10** | **Description** |
| --- | --- |
| C00-C97^1^ | Malignant neoplasms |
| D00-D09^1^ | In situ neoplasms |
| D45 | Polycythaemia vera |
| D46.0 | Myeolodysplastic syndromes: Refractory anaemia without ring sideroblasts, so stated |
| D46.1 | Myeolodysplastic syndromes: Refractory anaemia with ring sideroblasts |
| D46.2 | Myeolodysplastic syndromes: Refractory anaemia with excess of blasts (RAEB) |
| D46.9 | Myeolodysplastic syndrome, unspecified |

^1^ ICD10 codes excluded within C00-C97, as well as within D00-D09, are specified below.

#### Codes excluded within C00-C97 (malignant neoplasms):

| **ICD10** | **Description** |
| --- | --- |
| C08.8 | Malignant neoplasm: Overlapping lesion of major salivary glands |
| C15.0 | Malignant neoplasm: Cervical part of oesophagus |
| C15.1 | Malignant neoplasm: Thoracic part of oesophagus |
| C15.2 | Malignant neoplasm: Abdominal part of oesophagus |
| C26.8 | Malignant neoplasm: Overlapping lesion of digestive system |
| C39.8 | Malignant neoplasm: Overlapping lesion of respiratory and intrathoracic organs |
| C41.8 | Malignant neoplasm: Overlapping lesion of bone and articular cartilage |
| C44 (C44.0-C44.9) | Other malignant neoplasms of skin |
| C46.8 | Kaposi sarcoma of multiple organs |
| C72.8 | Malignant neoplasm: Overlapping lesion of brain and other parts of central nervous system |
| C76.7 | Malignant neoplasm: Other ill-defined sites |
| C80.9 | Malignant neoplasm, primary site unspecified |
| C82.7 | Other types of follicular lymphoma |
| C84.5 | Other mature T/NK-cell lymphomas |
| C84.8 | Cutaneous T-cell lymphoma, unspecified |
| C85.7 | Other specified types of non-Hodgkin lymphoma |
| C88.7 | Other malignant immunoproliferative diseases |
| C91.7 | Other lymphoid leukaemia |
| C91.8 | Mature B-cell leukaemia Burkitt-type |
| C92.7 | Other myeloid leukaemia |
| C92.8 | Acute myeloid leukaemia with multilineage dysplasia |
| C93.7 | Other monocytic leukaemia |
| C94.7 | Other specified leukaemias |
| C95.7 | Other leukaemia of unspecified cell type |
| C96.7 | Other specified malignant neoplasms of lymphoid, haematopoietic and related tissue |
| C96.8 | Histiocytic sarcoma |
| C97 | Malignant neoplasms of independent (primary) multiple sites |

#### Codes excluded within D00-D09 (in situ neoplasms):

| **ICD10** | **Description** |
| --- | --- |
| D04  (D04.0-D04.9) | Carcinoma in situ of skin |
| D05.7 | Other carcinoma in situ of breast |
| D09.7 | Carcinoma in situ of other specified sites |

## Covariates

This table presents the covariates used in the statistical modelling:

| **Covariate** | **Description** |
| --- | --- |
| Baseline LDL-C | The most recent low-density lipoprotein cholesterol (LDL-C) values within two years prior to index was selected. |
| Time-varying LDL-C | The most recent LDL-C prior to each given time-split segment in the clone-censor-weight analysis was selected. |
| Ongoing lipid-lowering treatment (LLT) | Ongoing treatment with LLT was defined as a filled pharmacy dispensation within 12 months prior to index (including the index date). |
| Time-varying LLT treatment | Time-varying treatment was defined as a pharmacy dispensation where medical supply overlapped with the start of each given time-split segment in the clone-censor-weight analysis. |
| Comorbid conditions | All comorbidities at baseline were based on diagnosis codes recorded at any time up until index. Diagnosis codes recorded in any healthcare level and in any diagnosis position were included.  The following comorbid conditions were included:  **Stroke** (ICD10: I60-I66 and G45)  **ASCVD** (ICD10: I20-I25, I70, I71, I72, I73.1, I73.9, I74, I77.3, I77.6, I77.8, I79, I60-I66 and G45)  **Heart failure** (ICD10: I50, I11.0, I13.0, I13.2)  **Pancreatitis** (ICD10: K85)  **Hypertension** (ICD10: I10)  **Obesity** (ICD10: E66)  **Diabetes mellitus** (ICD10: E10, E11)  **Dyslipidemia** (ICD10: E78)  **Muscle related disorders** (ICD10: M60-M63)  **COPD** (ICD10: J44)  **Hepatic disorders** (ICD10: K70-K77)  **CKD** (ICD10: E10.2, E11.2, E12.2, E13.2, E14.2, N08.3, N17-N19, I12.0, I12.0, I13.1, I13.9, Z49.1, Z40.2, Z49, Z99.2) |
| MI and stroke – time-varying | Time-varying covariates stroke and MI was based on primary diagnoses in inpatient care after MI discharge up until the start of a given time-split segment. Only MI events after 30 days from index were counted for time-varying MI. |

# Supplementary Figures

## Supplementary Figure 1. Incidence of the six negative control outcomes

## Supplementary Figure 2. Comparison in covariate balance between truncated and non-truncated weights for the NCO of fractures

**Truncated weights (as shown in the corresponding figure from the main manuscript)**


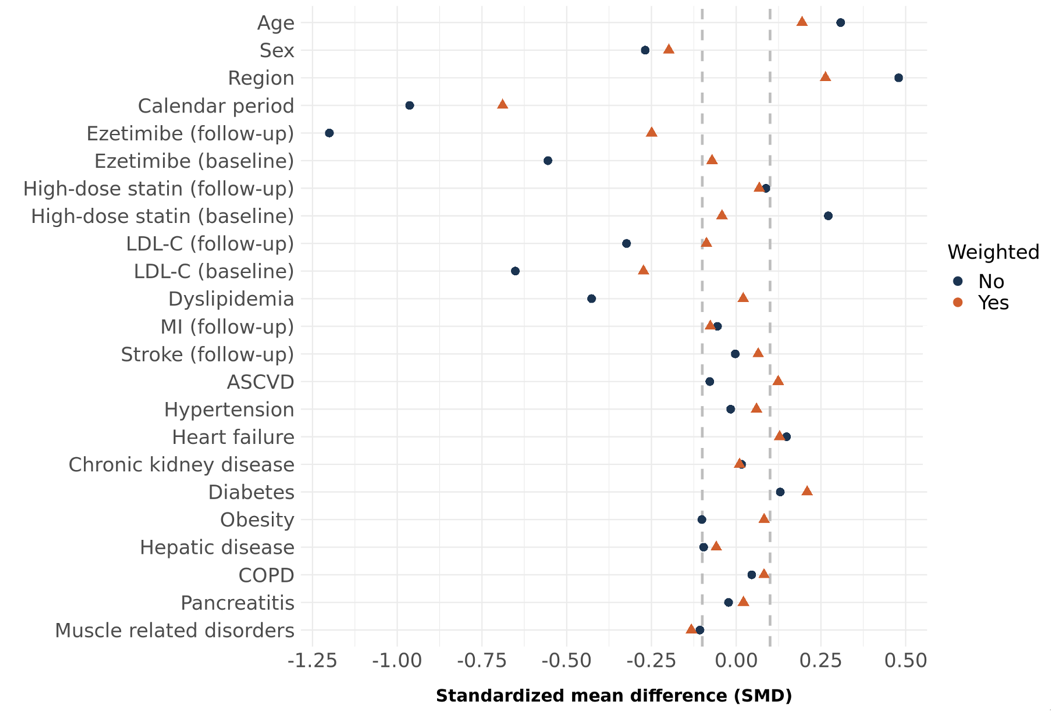


**Non-truncated weights**

**
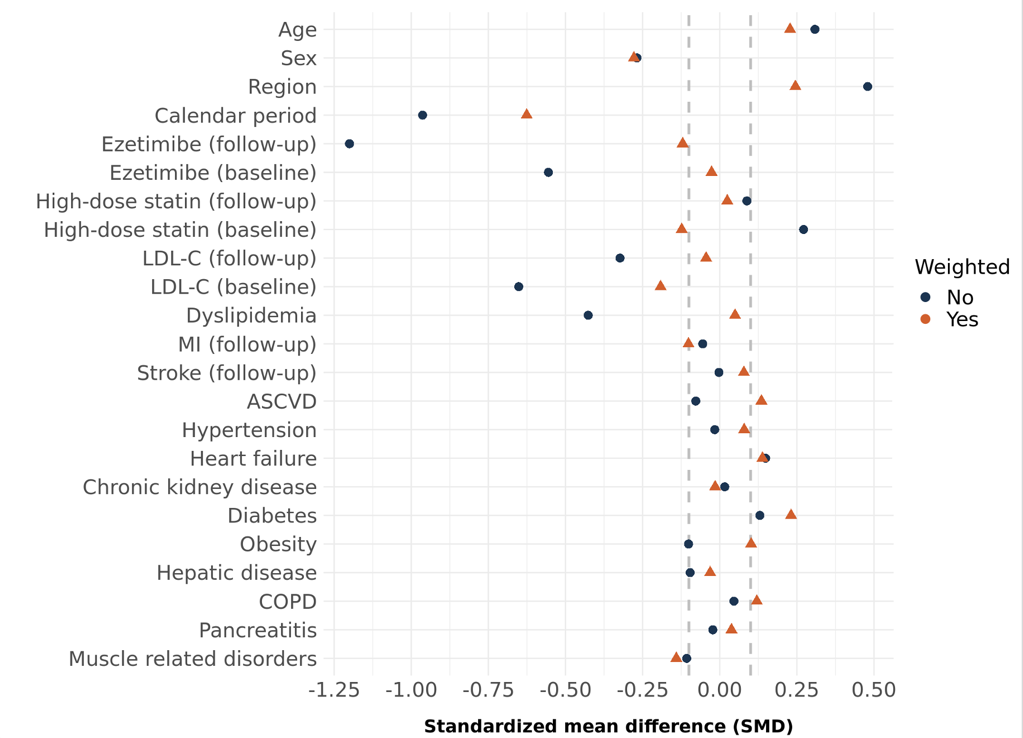
**

*Supplementary Figure 2 illustrates covariate balance before and after the application of weights. The upper panel shows results from the primary weighting strategy using truncated weights, while the lower panel presents the sensitivity analysis applying raw (non-truncated) weights.*

Supplementary Figure 3. Effect estimates and covariate balance for the NCO: fracture in the secondary study sample

Hazard ratio: 1.06 (95% CI: 0.70-1.35)

Risk difference: 0.0025 (95% CI: -0.023;0.024)

**Weighted Kaplan-Meier**

**
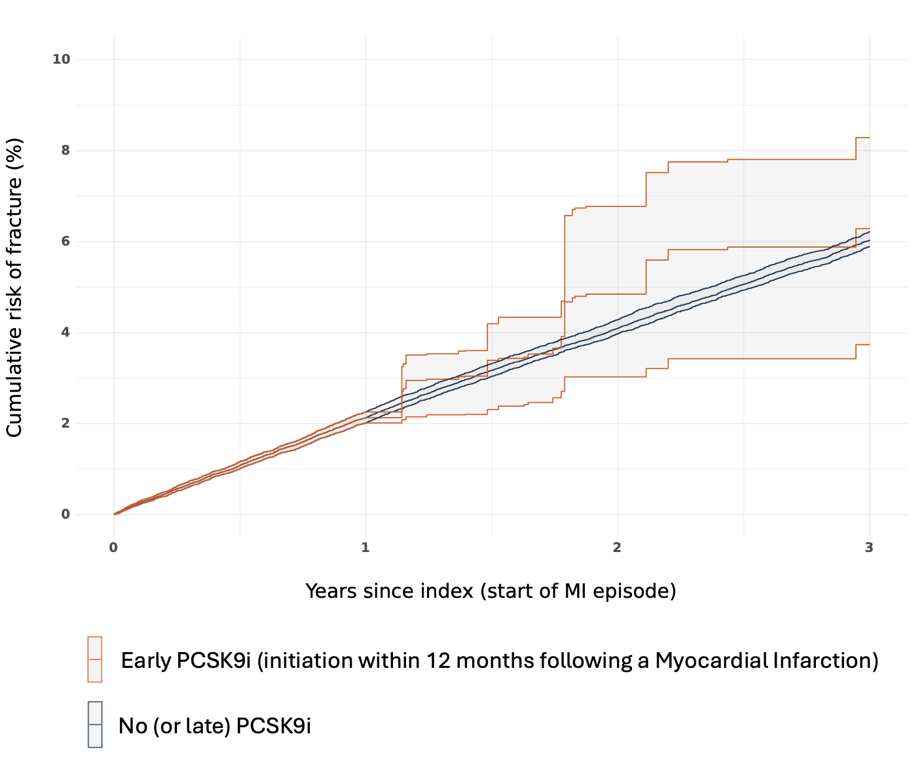
**

**Covariate balance**


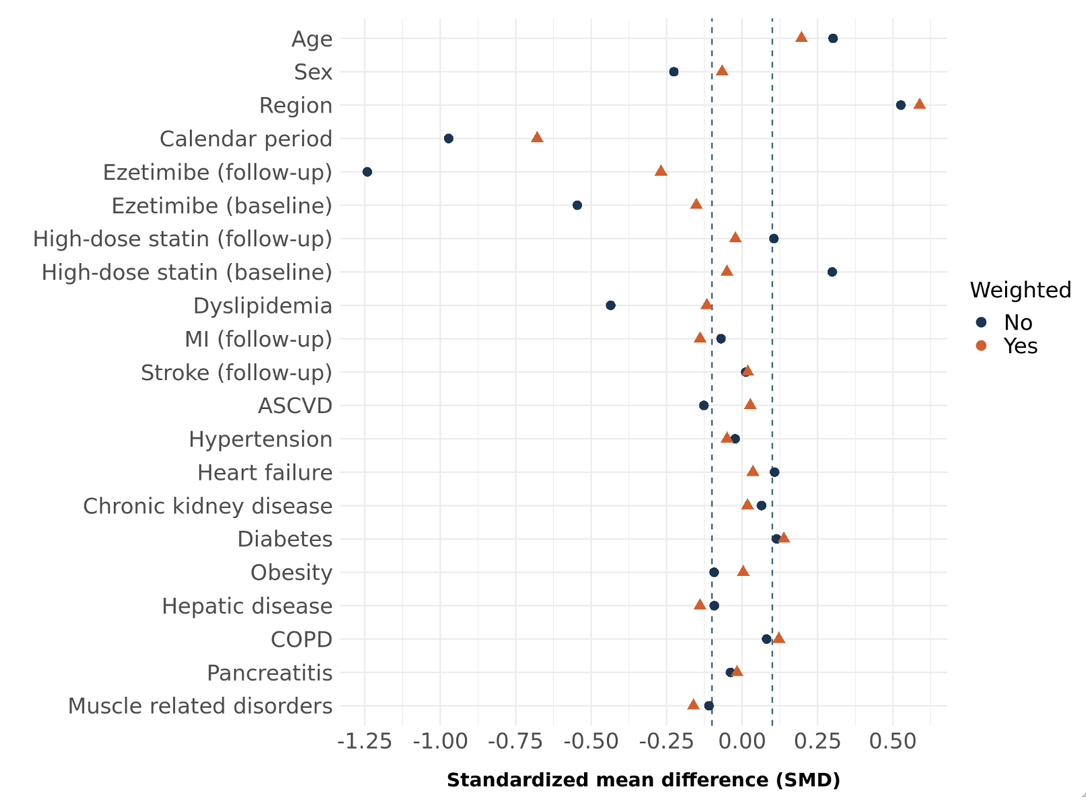


*Supplementary Figure 3 displays the effect estimates and covariate balance for the secondary study population, which includes a broader cohort of patients from all regions of Sweden.*

## Supplementary Figure 4. Sensitivity analysis using a simpler propensity score-weighted model

**Flow-chart**


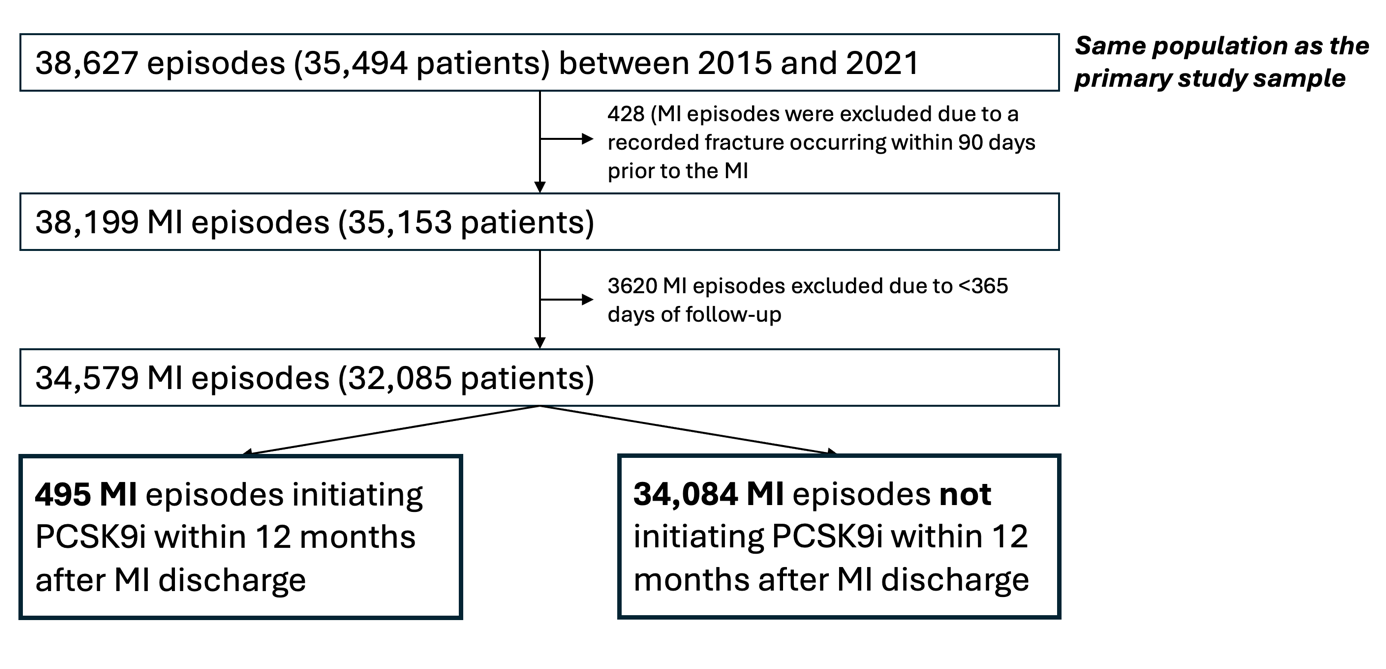


**Covariate balance**


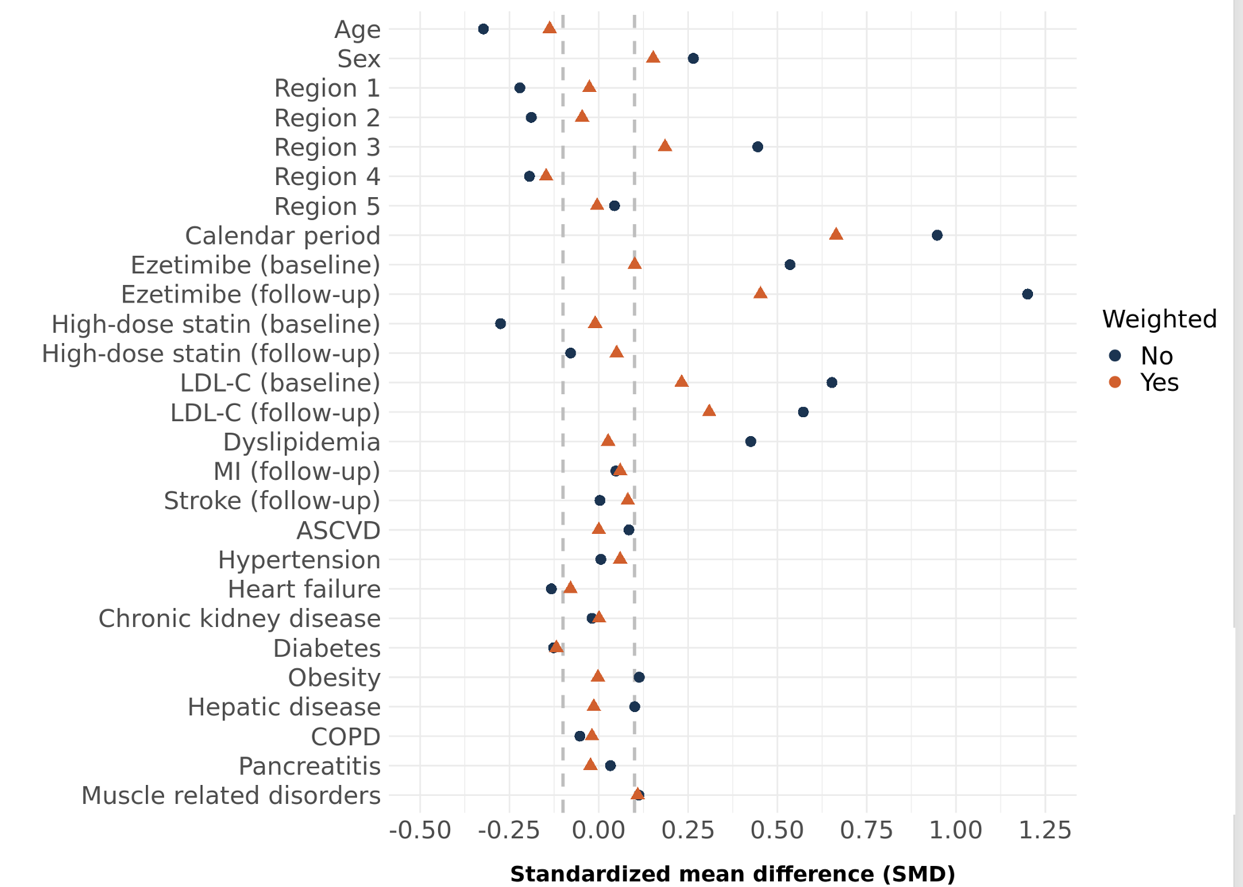


*This analysis applies a traditional propensity score-weighted approach, which is less complex than the clone-censor-weight method used in the primary analysis. The motivation of this sensitivity analysis was to assess whether similar covariate balance could be achieved with this simpler model.*

Supplementary Figure 5. Standardized mean differences between the early PCSK9i and no/late PCSK9i treatment strategies at 0, 3, 6, 9, and 12 months after index, with and without inverse probability of censoring weighting (weights truncated at the 99.9^th^ percentile).

*
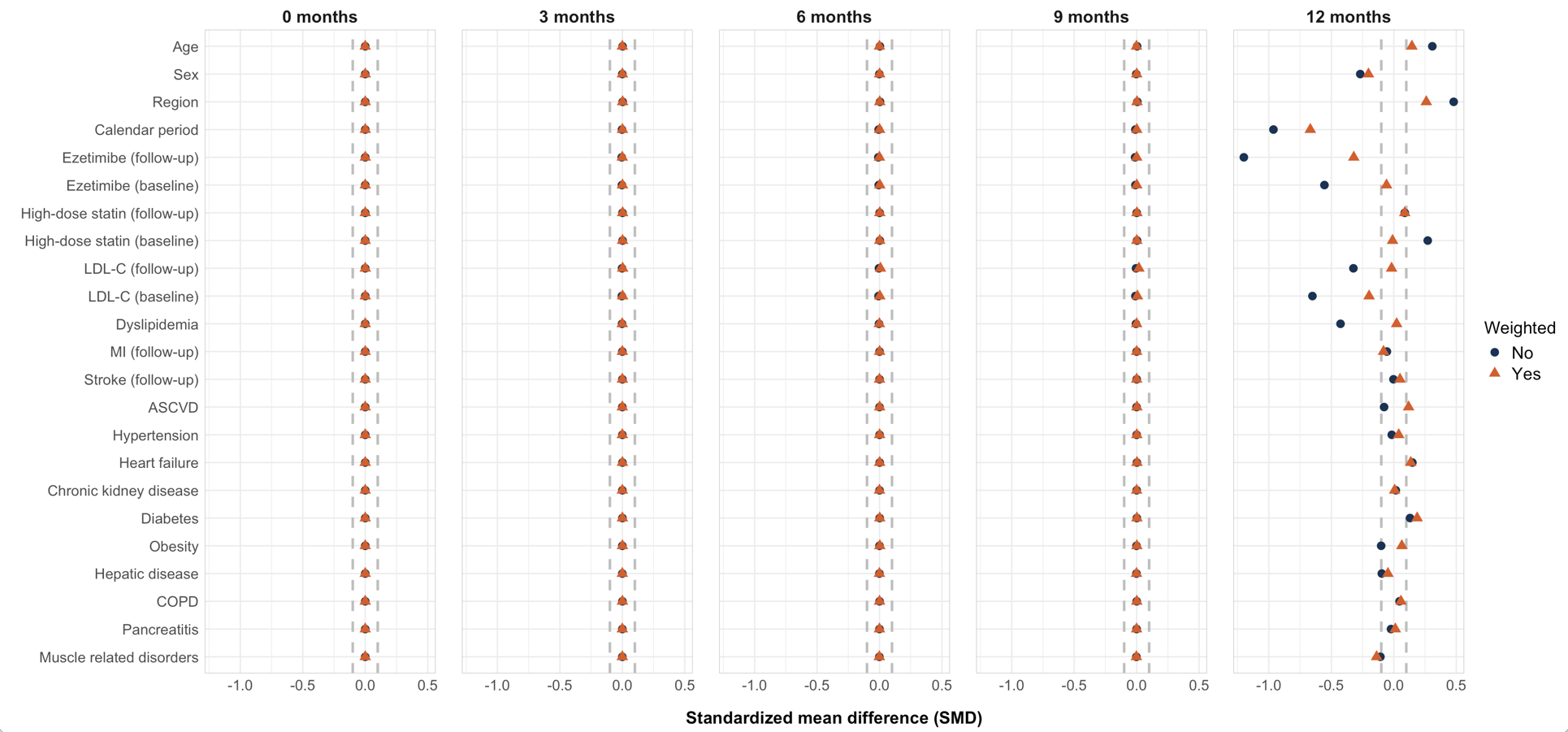
*

In the clone-censor-weight framework, all individuals are cloned into both treatment strategies at baseline, resulting in perfect covariate balance at month 0. As clones that deviate from their assigned strategy are progressively censored, imbalance emerges. The majority of censoring in the early PCSK9i arm occurs at 12 months, when clones that did not initiate treatment within 12 months are censored (i.e. when all informative censoring events had occurred). Dashed vertical lines indicate SMD thresholds of ±0.1.

## Supplementary Table 1. Distribution of inverse probability of censoring weights and effective sample size by treatment strategy, with and without truncation at the 99.9th percentile.

| **Treatment strategy** | **N** | **Mean** | **SD** | **Min** | **P1** | **P25** | **Median** | **P75** | **P99** | **P99,9** | **Max** | **Effective sample size (ESS)** |
| --- | --- | --- | --- | --- | --- | --- | --- | --- | --- | --- | --- | --- |
| Early PCSK9i (untruncated) | 479 | 52,2 | 87,1 | 1,2 | 1,8 | 10,3 | 21,3 | 50,4 | 405,1 | 715,1 | 817,4 | 127 |
| Early PCSK9i (truncated) | 479 | 45,1 | 56,7 | 1,2 | 1,8 | 10,3 | 21,3 | 50,4 | 214,0 | 214,0 | 214,0 | 186 |
| No/late PCSK9i (untruncated) | 33442 | 1,0 | 0,3 | 1,0 | 1,0 | 1,0 | 1,0 | 1,0 | 1,1 | 1,7 | 59,4 | 30240 |
| No/late PCSK9i (truncated) | 33442 | 1,0 | 0,3 | 1,0 | 1,0 | 1,0 | 1,0 | 1,0 | 1,1 | 1,7 | 59,4 | 30240 |

Weights were evaluated at the first weekly interval after all informative censoring had occurred (i.e. after 12 months). Truncation was applied at the 99.9th percentile of all weights across both strategies. The effective sample size (ESS) was calculated using the Kish approximation: ESS = (Σwᵢ)² / Σwᵢ². P1, P25, P75, P99, and P99.9 denote the 1st, 25th, 75th, 99th, and 99.9th percentiles, respectively.

## Supplementary Table 2. Bias-variance trade-off across weight truncation levels for the negative control outcome bone fracture

| **Truncation** | **HR** | **ESS (treated)** | **ESS (no treatment)** | **No. of covariates with SMD >0,1** | **Max abs SMD** | **Mean abs SMD** |
| --- | --- | --- | --- | --- | --- | --- |
| 95th | 1,01 | 479 | 33388 | 14 | 1,185 | 0,271 |
| 99th | 1,08 | 413 | 32829 | 15 | 1,065 | 0,226 |
| 99,9th | 0,96 | 186 | 30240 | 10 | 0,651 | 0,134 |
| Untruncated | 0,99 | 127 | 30240 | 11 | 0,587 | 0,126 |

HR: hazard ratio from a weighted Cox proportional hazards model comparing early versus no/late PCSK9i. ESS: effective sample size calculated using the Kish approximation (ESS = (Σwᵢ)² / Σwᵢ²). SMD: standardized mean difference. All diagnostics were evaluated at the first weekly interval after all informative censoring had occurred. Truncation was applied at the indicated percentile of all weights across both treatment strategies.
